# Pentraxin3 in Chronic Thromboembolic Pulmonary Hypertension: A New Biomarker for Screening from Remitted Pulmonary Thromboembolism

**DOI:** 10.1371/journal.pone.0113086

**Published:** 2014-11-20

**Authors:** Akira Naito, Nobuhiro Tanabe, Takayuki Jujo, Ayako Shigeta, Toshihiko Sugiura, Seiichiro Sakao, Keiichi Ishida, Koichiro Tatsumi

**Affiliations:** 1 Department of Respirology, Graduate School of Medicine, Chiba University, 1-8-1 Inohana, Chuo-Ku, Chiba 260-8670, Japan; 2 Department of Advanced Medicine in Pulmonary Hypertension, Graduate School of Medicine, Chiba University, 1-8-1 Inohana, Chuo-Ku, Chiba 260-8670, Japan; 3 Department of Cardiovascular Surgery, Graduate School of Medicine, Chiba University, 1-8-1 Inohana, Chuo-Ku, Chiba 260-8670, Japan; Keio University School of Medicine, Japan

## Abstract

**Background:**

Pentraxin3 (PTX3) is a protein, which has multifaceted effects on innate immunity, angiogenesis, and vascular remodeling then could be a disease marker of acute myocardial infarction, heart failure, vasculitis. In addition, PTX3 has been recognized as a biomarker for pulmonary arterial hypertension, however whether it is the case in chronic thromboembolic pulmonary hypertension (CTEPH) remains unclear. Therefore, we investigated whether PTX3 would be a useful biomarker for detecting CTEPH with respect to differentiation from stable pulmonary thromboembolism (PTE), in comparison to other biomarkers.

**Methods:**

Plasma PTX3 and brain natriuretic peptide (BNP) levels were measured in 70 patients with CTEPH at their first diagnostic right heart catheterization (CTEPH group) and in 20 patients with clinically stable PTE more than three months after the acute episode (control group). The levels of plasma C-reactive protein (CRP) and heart-type fatty acid-binding protein (H-FABP) were also analyzed to compare the diagnostic ability of these biomarkers.

**Results:**

The mean level of PTX3 (ng/mL) was significantly higher in the CTEPH group than in the control group (5.51±4.53 versus 2.01±0.96, respectively), and PTX3 levels had mild negative correlation with cardiac output. BNP levels were also higher in the CTEPH group and better correlated with pulmonary hemodynamics than PTX3. However, a receiver operating characteristic (ROC) curve showed PTX3 levels were better for detecting CTEPH, and could detect CTEPH patients with less severe pulmonary hemodynamics and low plasma BNP levels. There was no significant increase in CRP and H-FABP levels in the CTEPH patients.

**Conclusions:**

Plasma PTX3 level was the most sensitive biomarker of CTEPH. Although plasma PTX3 levels did not correlate with the severity of the pulmonary hemodynamics compared to BNP, high levels in clinically stable patients following PTE should prompt a further work-up for CTEPH, which may lead to an early diagnosis.

## Introduction

Chronic thromboembolic pulmonary hypertension (CTEPH) is a form of pulmonary hypertension caused by persistent thromboemboli of the pulmonary arteries [1]. It can be treated by pulmonary endarterectomy (PEA), provided that the thrombi are surgically accessible [2]. However, CTEPH can be difficult to treat if the thrombi are limited to peripheral pulmonary arteries, or if peripheral vascular remodeling has occurred, similar to the pathobiology in patients with idiopathic pulmonary arterial hypertension (PAH) [3], [4], [5], [6]. Few patients with acute pulmonary thromboembolism (PTE) develop CTEPH [7], but early diagnosis of this rare, refractory disease could improve prognosis. Currently, brain natriuretic peptide (BNP) is used widely as a biomarker of CTEPH similar to its use for chronic heart failure [8], [9]. It has also been reported that C-reactive protein (CRP) and heart-type fatty acid-binding protein (H-FABP) are biomarkers of CTEPH [10], [11].

Pentraxin3 (PTX3) is a novel attractive protein, belongs to the pentraxin family, which includes CRP. It is secreted locally by monocytes, macrophages, dendritic cells, endothelial cells, smooth muscle cells and renal mesangial cells in response to stimulation by interleukin (IL)-1β, tumor necrosis factor (TNF)-α, whereas CRP is produced by the liver [12]. PTX3 binds to several bacteria and viruses as pattern recognition molecule, activate the classical complement pathway by bindings to C1q [13]. Unlike CRP, PTX3 binds to several ligands other than C1q, including fibroblast growth factors (FGF)-2 (blood vessel repair/remodeling) [14], P-selectin (regulation of leukocyte recruitment, blood vessel inflammation) [15], [16], and TNF-α-stimulated gene6 (extracellular matrix organization) [16]. PTX3 is also reported to upregulate tissue factor expression in endothelial cells and play a role in thrombogenesis [17]. Any function described above might elevate PTX3 levels in angina pectoris [18], acute myocardial infarction [19], [20], heart failure [21], Takayasu arteritis [22], vasculitis [23], sepsis/systemic inflammatory response syndrome [24], and other infections [25].

Recently it was shown that PTX3 could reflect any pathophysiological aspect for PAH, especially in patients with connective tissue disease [26]. However, whether PTX3 could be one of the biomarkers in patients with CTEPH remains unclear. The purpose of this study was to investigate whether PTX3 would be a useful biomarker compared to other biomarkers for detecting CTEPH especially with respect to differentiation from clinically stable status after acute episode of PTE. We also examined the relationship between PTX3 levels and pulmonary hemodynamics in patients with CTEPH.

## Materials and Methods

### Ethical statement

The study protocol was approved by the institutional review board of Chiba University (approval number 1248), and written informed consent was obtained from all participating patients.

### Subjects

Plasma PTX3 levels were measured in 72 patients with CTEPH (CTEPH group) who were referred to Chiba University Hospital, Chiba, Japan, from 2001 to 2013. The diagnosis of CTEPH was confirmed as previously reported [27]. In brief, all patients underwent computed tomography (CT) scans, lung ventilation–perfusion scans, right-heart catheterization (RHC) and pulmonary angiography. The mean pulmonary arterial pressure (mPAP) of all participants was measured by RHC, and cardiac output (CO) and cardiac index (CI) by the thermo-dilution method. CTEPH was defined as an mPAP ≥25 mmHg with a normal wedge pressure in patients with a history of PTE and dyspnea for ≥6 months while on effective anticoagulation [5]. Two patients were excluded due to comorbid infectious diseases (lung abscess and non-tuberculous mycobacterial infection), and a total of seventy patients were included in the CTEPH group.

Forty-nine of the 70 patients underwent pulmonary endarterectomy (PEA). In nine of these patients, we also analyzed plasma PTX3 levels and RHC data one year after the operation.

The control group consisted of 20 patients with PTE (diagnosed by enhanced chest CT) who had obtained symptom remission while on warfarin maintenance therapy.

Plasma BNP but not N-terminal proBNP was analyzed in the same patients who had their PTX3 measured. The levels of plasma high-sensitive CRP and H-FABP were also analyzed in the 57 patients in the CTEPH group and 13 patients in the control group to compare the diagnostic ability of these biomarkers.

### Blood sampling and biomarker assays

Blood sampling was performed either at the first diagnostic RHC in the CTEPH group or during a routine medical consultation more than three months after the acute PTE episode in the control group. All the subjects were treated with warfarin. Sixteen CTEPH patients were given additional PAH-specific treatment (either epoprostenol, a phosphodiesterase-5 inhibitor, or an endothelin receptor antagonist). All blood sampling occurred prior to the administration of morning medications.

Plasma PTX3 levels were measured using an enzyme-linked immunosorbent assay kit (Perseus Proteomics Inc. Tokyo, Japan) and plasma H-FABP levels by a latex agglutination immunoassay kit (LIBLIA H-FABP, DS Pharma Biomedical Co. Ltd., Osaka, Japan). Assays were performed by SRL Inc. Tokyo, Japan.

### Statistical analysis

All the statistical analyses were performed with commercially available software (JMP 10.0.2, Japanese version, SAS Institute Tokyo, Japan). Results are expressed as mean ± standard deviation (SD) for continuous variables and as number and percentage for categorical variables unless otherwise indicated. The clinical characteristics of the CTEPH and PTE follow-up groups were compared using Student's *t*-test. Biomarkers including PTX3 were compared in the two groups using the Mann-Whitney test, while correlations between the biomarkers and hemodynamic parameters were assessed by the Spearman's rank correlation coefficient. The comparisons of hemodynamic parameters and biomarkers between pre and post PEA and between patients with or without PAH-specific treatment were assessed by the Wilcoxon matched-pairs signed-rank test and Mann-Whitney test. Receiver operating characteristic (ROC) curves were used to determine the optimal threshold PTX3 value for the diagnosis of CTEPH, and areas under ROC curves (AUC_ROC_) and 95% confidence intervals (CI) were calculated to compare the diagnostic efficacies.

## Results

### Clinical characteristics of patients with CTEPH

The clinical characteristics of patients with CTEPH are summarized in Table 1. The mean age of the 70 patients with CTEPH (16 men and 54 women) and the 20 control subjects (3 men and 17 women) was 59.0±11.4 and 63.0±15.4 years, respectively. All the patients were Japanese. The mean disease duration for patients with CTEPH, defined as the period of time from the onset of symptoms to diagnostic RHC, was 38.7±46.8 months. The mean disease duration for control patients, defined as the period of time from the onset of acute PTE to blood sampling, was 55.1±44.2 months. Patients with CTEPH weighed significantly less than controls (56.3±11.2 kg vs. 64.7±9.42 kg, *p* = 0.03). No significant differences in systemic blood pressure, triglycerides, total cholesterol or hemoglobin A1c (HbA1c) were observed between the two groups. Several patients had hypertension and hyperlipidemia, but none of the recruited subjects had any apparent severe comorbidity such as non-tubeculous mycobacterial infection, splenectomy, inflammatory bowel disease, cancer, or thyroid disease. One patient with CTEPH had a history of rheumatoid arthritis.

**Table 1:** Clinical characteristics of patients.

	CTEPH	Acute PTE follow-up	*p* value
	group	(Control)	
Number of patients	70	20	
Age (y)	59.0±11.4	63.0±15.4	n.s.
Sex (M:F)	16∶54	3∶17	n.s.
Height (cm)	157.9±10.0	154.5±6.26	n.s.
Weight (kg)	56.3±11.2	64.7±9.42	0.028†
Systolic blood pressure (mmHg)	125.4±19.3	128.3±18.0	n.s.
Diastolic blood pressure (mmHg)	77.1±14.1	70.7±9.25	n.s.
Disease duration (month)	38.7±46.8	55.1±44.2	n.s.
Triglyceride (mg/dL)	142.2±87.0	110.0±50.3	n.s.
Total cholesterol (mg/dL)	203.1±38.5	202.1±22.0	n.s.
HbA1c (%)	5.53±0.54	5.36±0.37	n.s.

Data are presented as either mean ± SD or actual value.

HbA1c: Hemoglobin A1c.

† : p<0.05.

### Plasma PTX3 and other biomarkers

**Figure 1:**
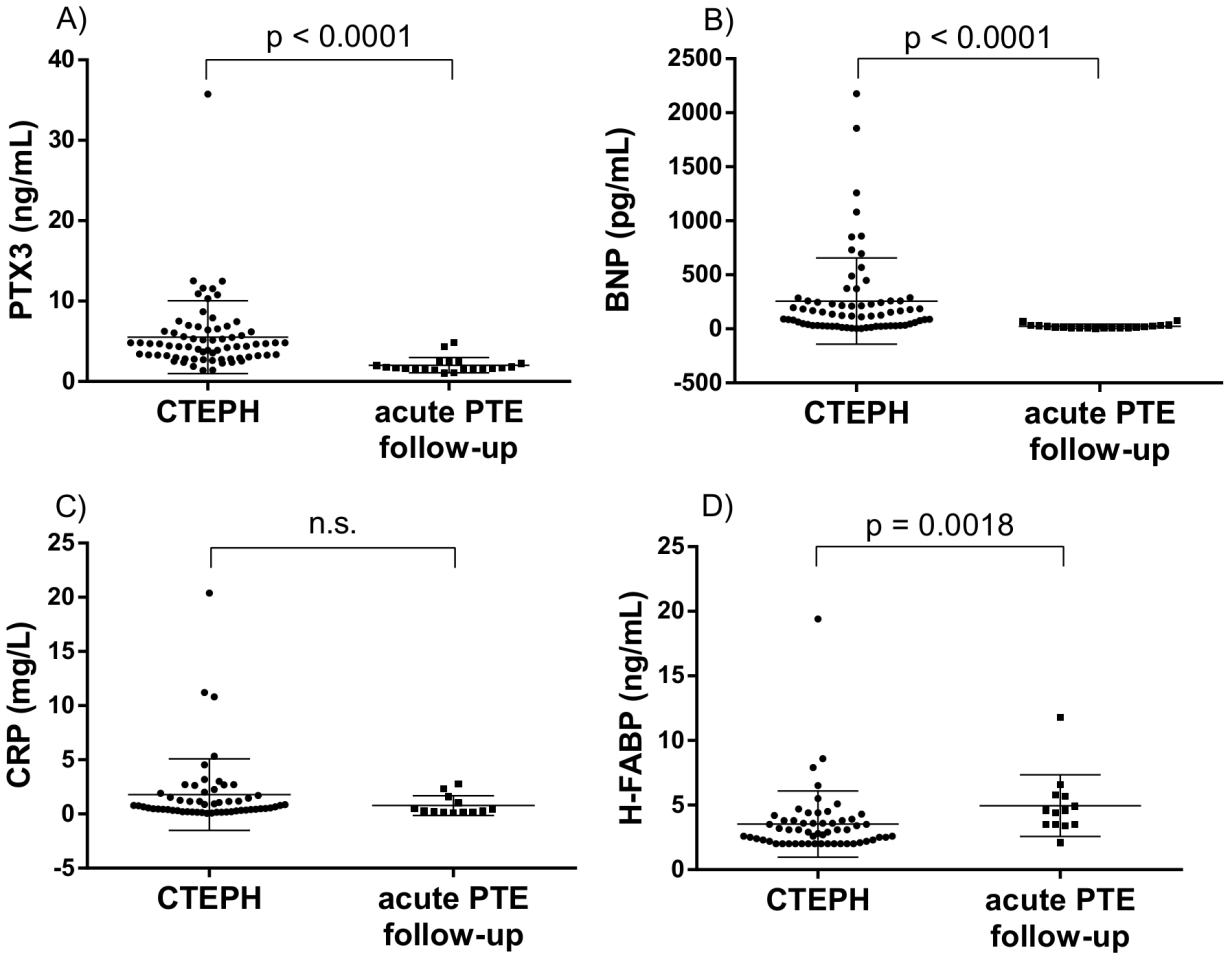
Plasma pentraxin3 (PTX3) and other biomarker's concentration in patients with CTEPH. A) PTX3 (ng/mL), B) BNP (pg/mL), C) CRP (mg/L), and D) H-FABP. Plasma PTX3 and BNP levels were higher in patients with CTEPH than in controls. The CTEPH patients tended to have higher plasma CRP levels than the control patients, although this difference was not statistically significant. Both groups had a low plasma H-FABP level which is considered to be almost normal [28], although control patients had statistically higher H-FABP level than CTEPH patients.

Plasma PTX3 levels were higher in patients with CTEPH than in controls [5.51±4.53 ng/mL (range, 1.39 to 35.7) vs. 2.01±0.96 ng/mL (range, 1.06 to 4.84), *p*<0.0001]. Plasma BNP levels were also higher in patients with CTEPH than in controls (257.3±398.3 pg/mL vs. 23.0±20.1 pg/mL, *p*<0.0001). Although the CTEPH patients tended to have higher plasma CRP levels than control patients, but this difference was not statistically significant between the two groups (CTEPH group, 1.78±3.30 mg/L vs. control group, 0.77±0.90 mg/L, *p* = 0.1075). Both groups had low plasma H-FABP levels that were considered almost normal [28], although control patients had statistically higher levels than the CTEPH patients (CTEPH group, 3.53±2.55 ng/mL vs. control group, 4.95±2.38 ng/mL, *p* = 0.0018) (Figure 1). Correlation analyses of the biomarkers showed there was a weak relationship between PTX3 and BNP (ρ = 0.3015, *p* = 0.0125), and H-FABP and BNP (ρ = 0.3389, *p* = 0.0114), whereas there was no significant association between PTX3 and CRP/H-FABP.

### Plasma PTX3 and pulmonary hemodynamics

**Figure 2:**
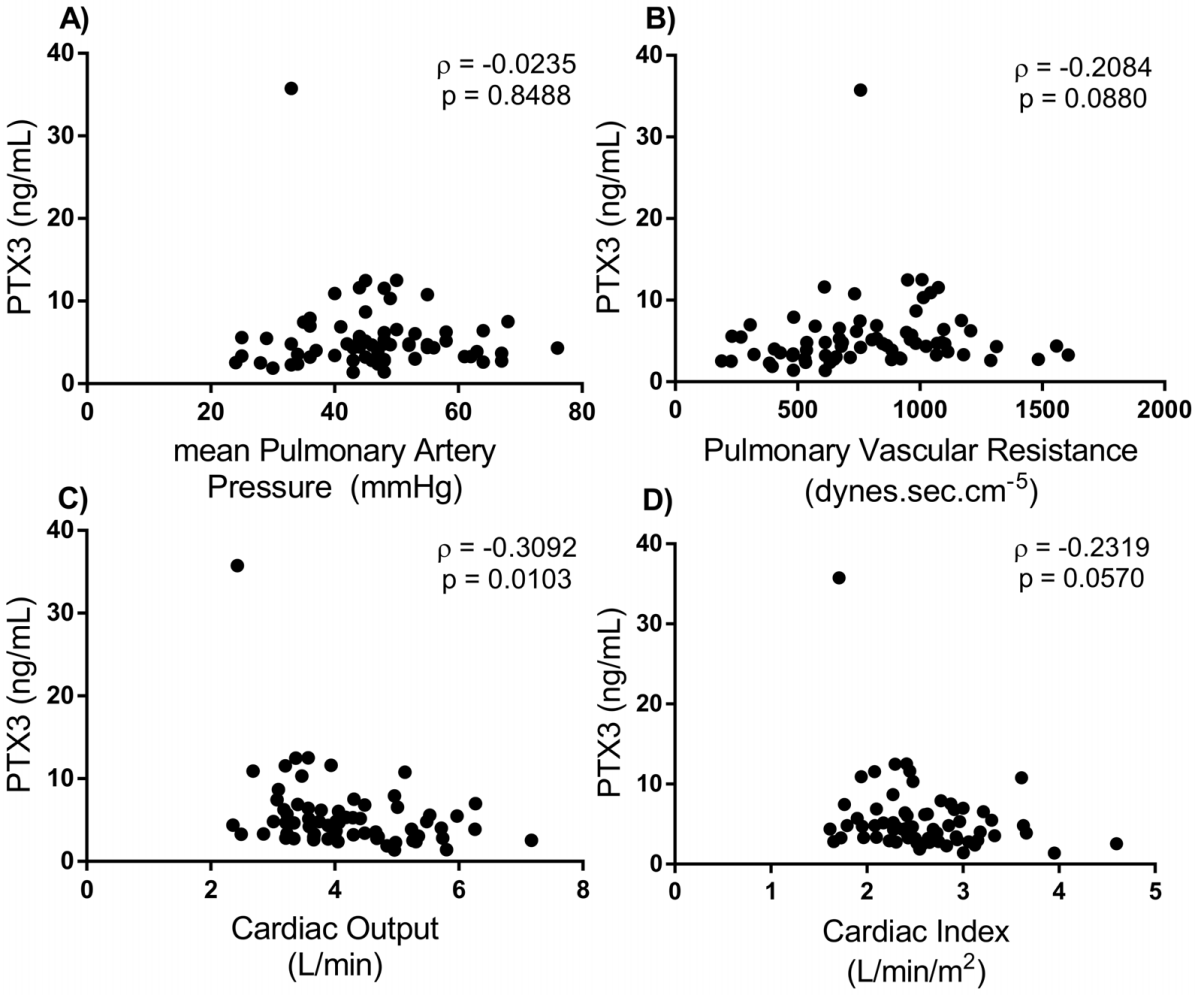
Scatter diagram data on the correlation between PTX3 and pulmonary hemodynamic patameters. A) Mean pulmonary arterial pressure (mmHg), B) Pulmonary vascular resistance (dynes.sec.cm^−5^), C) cardiac output (L/min), and D) cardiac index (L/min/m^2^). We found a mild negative correlation between PTX3 levels and cardiac output (CO) in patients with CTEPH. There was no significant correlation between PTX3 and other hemodynamic parameters.

As shown in Figure 2 and Table 2, we found a mild negative correlation between PTX3 levels and cardiac output (CO) in the patients with CTEPH. There was a considerably stronger degree of correlation between BNP levels and hemodynamic parameters than that observed for PTX3. There was no correlation between CRP/H-FABP and hemodynamic parameters. In the 49 CTEPH patients who underwent PEA, no correlation was observed between PTX3 levels and the Jamieson classification [type1 (n = 29), 5.39±2.67 ng/mL; type2 (n = 13), 6.60±9.09 ng/mL; type3 (n = 7), 5.69±2.53 ng/mL; type4 (n = 0), Steel-Dwass test.] [29].

**Table 2:** Correlation coefficient between biomarker levels and pulmonary hemodynamic parameters.

Factors	ρvalue	*p* value	
PTX3			
Mean pulmonary artery pressure (mmHg)	−0.0235	0.8488	
Pulmonary vascular resistance (dyne.sec.cm^−5^)	0.2084	0.0880	
Cardiac output (L/min)	−0.3092	0.0103	†
Cardiac index (L/min/m^2^)	−0.2319	0.0570	
BNP			
Mean pulmonary artery pressure (mmHg)	0.4508	0.0001	‡
Pulmonary vascular resistance (dyne.sec.cm^−5^)	0.6998	<0.0001	‡
Cardiac output (L/min)	−0.6366	<0.0001	‡
Cardiac index (L/min/m^2^)	−0.6185	<0.0001	‡
CRP			
Mean pulmonary artery pressure (mmHg)	0.0384	0.7805	
Pulmonary vascular resistance (dyne.sec.cm^−5^)	0.1342	0.3288	
Cardiac output (L/min)	−0.2634	0.0520	
Cardiac index (L/min/m^2^)	−0.1957	0.1521	
H-FABP			
Mean pulmonary artery pressure (mmHg)	0.0789	0.5669	
Pulmonary vascular resistance (dyne.sec.cm^−5^)	0.2522	0.0633	
Cardiac output (L/min)	−0.2566	0.0586	
Cardiac index (L/min/m^2^)	−0.2177	0.1103	

† : p<0.05;

‡ : p<0.01.

### Treatment effect on plasma PTX3

Plasma PTX3 levels and RHC data were evaluated one year after PEA in nine patients with CTEPH (Table 3). In these patients, no significant differences in the plasma level of PTX3 or other biomarkers were observed with the exception of CRP before and after one year of PEA, despite an improvement in mPAP and PVR. There was also no correlation between post-operative PTX3 and post-operative hemodynamic parameters including CO. We also observed no significant difference in PTX3, the level of other biomarkers, and mPAP or PVR between patients treated with PAH-specific therapies and patients who were not so treated (PTX3: 5.94±3.52 ng/mL vs. 5.38±4.81 ng/mL, respectively, *p* = 0.405).

**Table 3:** Effects of pulmonary endarterectomy (PEA) on hemodynamic parameters, PTX3, and other biomarkers.

Factor	Before PEA	After PEA	*p* value	
Mean pulmonary artery pressure (mmHg)	43.3±11.0	23.1±4.34	0.0078	‡
Pulmonary vascular resistance (dyne.sec.cm^−5^)	729.9±311.6	290.2±123.5	0.0039	‡
Cardiac output (L/min)	3.80±0.55	4.04±0.47	0.2500	
Cardiac index (L/min/m^2^)	2.47±0.45	2.57±0.24	0.3789	
Stroke volume index	36.4±8.66	36.3±2.84	0.8438	
PTX3 (ng/mL)	3.82±0.92	4.04±2.24	0.570	
BNP (pg/mL)	92.0±83.7	36.3±20.5	0.425	
CRP (mg/L)	3.32±4.40	0.57±0.34	0.0117	†
H-FABP (ng/mL)	2.44±1.15	2.77±1.87	0.2500	

Wilcoxon matched-pairs signed-rank test.

† : p<0.05;

‡ : p<0.01.

### Receiver operating characteristic (ROC) curves for detecting CTEPH

**Figure 3:**
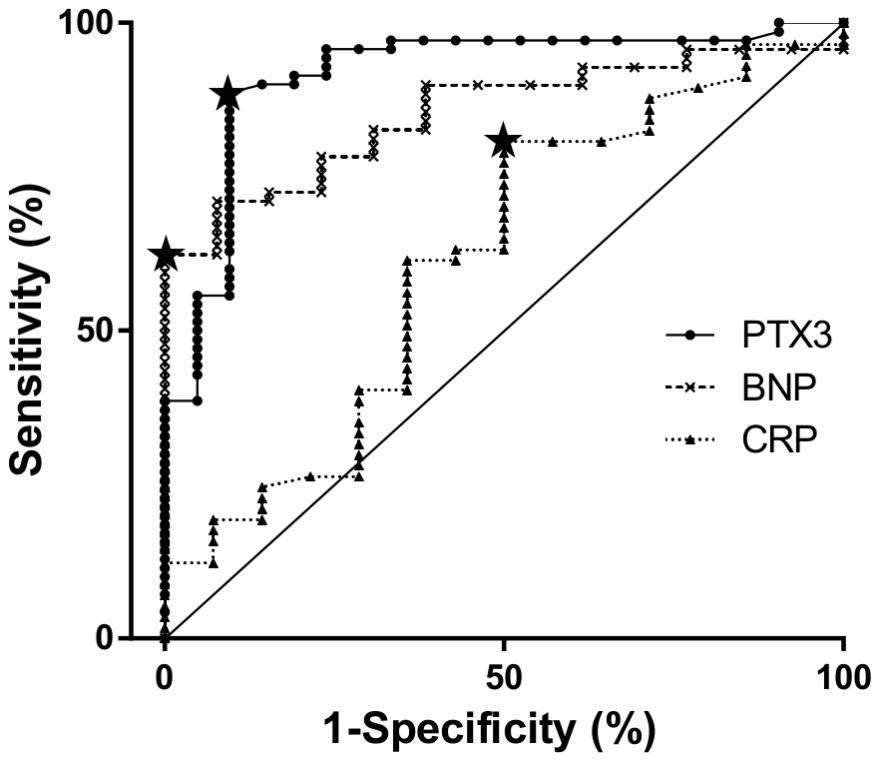
AUC_ROC_ for PTX3, BNP, and CRP in patients with CTEPH. Receiver operating characteristic (ROC) curves of PTX3, BNP, and CRP. The area under the ROC curve (AUC_ROC_) is 0.913 [0.837–0.990] for PTX3, 0.863 [0.793–0.945] for BNP, and 0.644 [0.4714–0.81074] for CRP. A PTX3 threshold of 2.63 ng/mL maximizes the sensitivity and specificity of CTEPH diagnosis (sensitivity 88.5% and specificity 90.5%).

The PTX3 levels in patients with or without CTEPH were analyzed to determine the predictive value of PTX3 for the diagnosis of CTEPH. The ROC curves suggest that PTX3 (area under the curve (AUC) 0.913 [95% CI 0.837–0.990]) has better sensitivity and specificity for predicting CTEPH than either BNP (AUC 0.863 [95% CI 0.793–0.945]) or CRP (AUC 0.6444 [95% CI 0.4714–0.8174]). We did not draw a ROC curve of H-FABP because the CTEPH patients did not have a statistically higher H-FABP level than that measured in the control patients. A PTX3 level>2.63 ng/mL was highly sensitive and specific for CTEPH in this patient population (sensitivity 88.5%, specificity 90.5%) (Figure 3). Comparing the PTX3 and BNP contingency table using a BNP value>35 pg/mL as the cut-off value [9], showed there were a few BNP-negative but PTX3-positive patients in the CTEPH group (Table 4). Interestingly, these patients had lower mPAP and PVR and higher CO and CI levels than patients who were positive for both BNP and PTX3. When we used a BNP value>23.4 pg/mL as the cut-off value, BNP levels had the same sensitivity as PTX3 (88.5%), but the specificity decreased to 65%.

**Table 4:** Contingency table for PTX3 and BNP levels.

	PTX3 ≥2.63 ng/mL	PTX3 <2.63 ng/mL	Total
BNP ≥35 pg/mL	50 (49)	3 (1)	53 (50)
BNP <35 pg/mL	13 (12)	24 (8)	37 (20)
Total	63 (61)	27 (9)	90 (70)

Values indicate the number of subjects including CTEPH patients and controls. Values within () indicate the number of CTEPH patients.

## Discussion

To our knowledge, this is the first study to demonstrate elevated plasma PTX3 levels in patients with CTEPH. In addition, we found that PTX3 levels showed mild negative correlation with CO. No significant difference was observed in PTX3 levels before and after successful PEA, or between patients who were or were not treated with PAH-specific therapies. PTX3 levels had better sensitivity than BNP levels, although BNP levels showed considerably stronger correlations with pulmonary hemodynamic parameters. Compared to BNP, PTX3 could identify CTEPH patients with less severe pulmonary hemodynamics.

As described above, PTX3 is produced locally in the various cells in response to the stimulation by inflammatory cytokines. On the other hand, BNP is produced mainly in the cardiac ventricles by an increase in stretch and/or pressure [30]. It is thus expected that BNP levels will correlate with hemodynamic parameters, however, BNP levels paradoxically tend to miss the CTEPH patients with preserved right ventricular ejection fraction [31] or low pulmonary arterial pressure [32]. Lang et al. reported that inflammation not only contributes to the pathogenesis of CTEPH, but also to the development of the disease [1]. We speculated that PTX3 levels may be able to detect the early stage of CTEPH with inflammation before the disease develops to mechanically severe pulmonary hypertension. In other words, PTX3 may be a sensitive biomarker that indicates the “existence of inflammation behind the disease” whereas BNP is a biomarker that reflects the severity of mechanical ventricle stress.

However, the question remains on the origin of elevated PTX3 levels. A previous study on patients with left heart failure reported a negative linear correlation between PTX3 levels and ejection fraction [21]. Leary et al. showed that higher PTX3 levels were associated with greater right ventricle mass and larger right ventricle end-diastolic volume in patients with atherosclerosis [33]. Our study revealed a mild correlation between PTX3 levels and CO in patients with CTEPH. It is therefore possible that PTX3 is derived from inflammation in the right ventricle. On the other hand, Zabini et al. reported an increase in inflammatory cytokine concentration in PEA tissue [34], so it is possible that the increase in PTX3 is also produced at the organized thrombus. We could not define which tissue is the main producer of PTX3, but we speculate that there may be a complex inflammatory interaction between organized thrombi, the peripheral pulmonary vasculature, and right heart ventricle muscle. However, further investigation is needed to clarify these relationships.

Regarding the clinical diagnostic efficacies, it has previously been reported that PTX3 level in the healthy population is 2.00 ng/mL [95% CI 1.95–2.04] [35]. That is similar to the PTX3 levels in the control group of this study (2.01 ng/mL [95% CI 1.55–2.46]), which consisted of patients with a history of PTE who had symptom improvement on warfarin therapy. This finding suggests that PTX3 may be a useful screening tool for identifying CTEPH even in patients with a history of PTE. For example, elevated plasma PTX3 levels in this patient population may prompt further work-up for CTEPH (i.e., echocardiogram, pulmonary ventilation–perfusion scans, RHC), which may lead to an early diagnosis. It should be noted, however, that elevated PTX3 levels have been observed in conditions other than pulmonary hypertension as described above, and careful interpretation of the data is required. Just for information, we also evaluated the PTX3 levels of three PTE patients in acute phase (in a week within the admission). They showed relatively high PTX3 levels (7.69, 3.46, and 1.63 ng/mL) (Table S1). We assume that patients with severe PTE in acute phase have also high PTX3 levels, and the elevated PTX3 level decreases as the disease gets relieved. The increased level of PTX3 in some CTEPH patients may be prolonged from the onset of acute PTE as pulmonary vasculature degeneration and/or right heart burden are prolonged. Hence, it may be difficult to distinguish the patients with severe PTE in acute phase from the patients with CTEPH.

Tamura et al. previously reported elevated levels of PTX3 in patients with PAH (idiopathic PAH, PAH associated with connective tissue disease and PAH associated with congenital heart disease) [26]. They found that PTX3 levels did not correlate with mPAP, PVR or BNP, which is similar to our findings (they did not report CO or CI values). Finally, they found a relatively low level of PTX3 in patients undergoing PAH-specific treatment and suggested that PTX3 may derive from pulmonary vascular degeneration. We found that neither PAH-specific treatment nor PEA has any significant effect on PTX3 levels. It is unclear why the two studies differ in this view, but they had a common suggestion that PTX3 levels may not simply reflect the severity of PAH as described above.

Our study had several limitations. First, the ROC curves may have been biased by the high prevalence of patients with CTEPH in our institute, which is considerably greater than the prevalence in most clinical settings. Second, we could not measure plasma PTX3 in a few patients who underwent PEA. There was no significant correlation between post-PEA PTX3 levels and post-PEA hemodynamic parameters, and there was also no significant difference between PTX3 levels measured pre- and post-PEA. The sample size is too small to evaluate these data. Third, this study was performed retrospectively at a single institution. An additional prospective, multicenter investigations are therefore required.

## Conclusion

In conclusion, this is the first report demonstrating elevated plasma PTX3 levels in patients with CTEPH. PTX3 level has better sensitivity than BNP level for detecting CTEPH patients, especially with less severe pulmonary hemodynamic parameters. High plasma PTX3 levels in clinically stable patients following PTE should prompt further work-up for CTEPH, which may lead to an early diagnosis.

## Supporting Information

Table S1 **Plasma PTX3 and BNP values in three acute PTE patients (in a week within the admission).** PTX3 levels of three PTE patients in acute phase (in a week within the admission). They showed relatively high PTX3 levels. In case 1 of the table, plasma PTX3 level decreased from 7.63 to 4.59 ng/mL in two weeks.
(DOCX)
