# Supplementary material for: Pentraxin3 in Chronic Thromboembolic Pulmonary Hypertension: A New Biomarker for Screening from Remitted Pulmonary Thromboembolism
Source: PLoS One. 2014 Nov 20;9(11):e113086. doi: 10.1371/journal.pone.0113086 (PMC4239022; doi:10.1371/journal.pone.0113086)
Supplement: Table S1 — Plasma PTX3 and BNP values in three acute PTE patients (in a week within the admission). PTX3 levels of three PTE patients in acute phase (in a week within the admission). They showed relatively high PTX3 levels. In case 1 of the table, plasma PTX3 level decreased from 7.63 to 4.59 ng/mL in two weeks. (DOCX) [file pone.0113086.s001.docx]

**Table S1: Plasma PTX3 and BNP values in three acute PTE patients (in a week within the admission)**

| Case | Sex | Age (y) | PTX3 (ng/mL) | BNP (ng/mL) | Comorbidities |
| --- | --- | --- | --- | --- | --- |
| 1. | Female | 82 | 7.63 | 91.6 | hypertension |
| 2. | Female | 41 | 3.46 | 40.5 | 2 months after the natural childbirth |
| 3. | Male | 42 | 1.63 | 10.8 | None (2 weeks after the plane boarding ) |

In case 1 of the table, plasma PTX3 level decreased from 7.63 to 4.59 ng/mL in two weeks.
